# Multi-objective optimized genomic breeding strategies for sustainable food improvement

**DOI:** 10.1038/s41437-018-0147-1

**Published:** 2018-09-27

**Authors:** Deniz Akdemir, William Beavis, Roberto Fritsche-Neto, Asheesh K. Singh, Julio Isidro-Sánchez

**Affiliations:** 1 Cornell Statistical Consulting Unit, Cornell University, Ithaca, NY USA; 2 Department of Agronomy, Iowa State University, Ames, IA USA; 3 Department of Genetics, “Luiz de Queiroz” Agriculture College, University of Sao Paulo, Sao Paulo, Brazil; 4 Agriculture & Food Science, Animal and Crop section, University College Dublin, Dublin, Ireland

**Keywords:** Plant breeding, Animal breeding, Inbreeding, Quantitative trait, Genetic markers

## Abstract

The purpose of breeding programs is to obtain sustainable gains in multiple traits while controlling the loss of genetic variation. The decisions at each breeding cycle involve multiple, usually competing, objectives; these complex decisions can be supported by the insights that are gained by applying multi-objective optimization principles to breeding. The discussion in this manuscript includes the definition of several multi-objective optimized breeding approaches within the phenotypic or genomic breeding frameworks and the comparison of these approaches with the standard multi-trait breeding schemes such as tandem selection, independent culling and index selection. Proposed methods are demonstrated with two empirical data sets and simulations. In addition, we have described several graphical tools that can aid breeders in arriving at a compromise decision. The results show that the proposed methodology is a viable approach to answer several real breeding problems. In simulations, the newly proposed methods resulted in gains larger than the methods previously proposed including index selection: Compared to the best alternative breeding strategy, the gains from multi-objective optimized parental proportions approaches were about 20–30% higher at the end of long-term simulations of breeding cycles. In addition, the flexibility of the multi-objective optimized breeding strategies were displayed with methods and examples covering non-dominated selection, assignment of optimal parental proportions, using genomewide marker effects in producing optimal mating designs, and finally in selection of training populations for genomic prediction.

## Introduction

There are two ways in which the action of a breeder can change the genetic properties of the population; the first by the choice of individuals to be used as parents, which constitutes selection (Allard 1999; Falconer et al. 1996) and the second by control of the way in which the parents are mated, which embraces inbreeding and cross-breeding (Akdemir and Sánchez 2016; Fernández et al. 2001; Kinghorn and Shepherd 1999; Pryce et al. 2012; Shepherd and Kinghorn 1998; Sun et al. 2013; Wright 1921). Selection means breeding from the “best” individuals whatever “best” might be (Allard 1999). The simplest form of selection is to choose individuals based on their own phenotypic values. Nevertheless, the breeding value (BV) of an individual is what influences the next generation. If breeders choose individuals to be parents according to their phenotypic values, their success in changing the population can be predicted only from knowledge of the degree of correspondence between phenotypic values and BVs (heritability) (Cockerham 1963; Dudley and Moll 1969; Holland et al. 2003).

Breeders have been selecting on the basis of phenotypic values since domestication of plants and animals. More recently, breeders have substantially used the pedigree-based prediction of genetic values for the genetic improvement of complex traits (Crossa et al. 2006; Gianola and Fernando 1986; Henderson 1984; Piepho et al. 2008).

The enhancements in high throughput genotyping (Lander et al. 2001; Margulies et al. 2005; Metzker 2010) have transformed breeding pipelines through marker-assisted selection (MAS) (Lande and Thompson 1990), marker-assisted introgression (Charcosset and Hospital 1997), marker-assisted recurrent selection (Bernardo and Charcosset 2006), and genomic selection (GS) (Meuwissen et al. 2001). The latter uses genome-wide markers to estimate the effects of all genes or chromosome positions simultaneously to calculate genomic estimated breeding values (GEBVs), which are used for the selection of individuals. This process involves the use of phenotypic and genotypic data to build prediction models that would be used to estimate GEBVs from genome wide marker data. It has been proposed that GS increases the genetic gains by reducing the generation intervals and also by increasing the accuracy of estimated BVs.

The economic value of the final product in a breeding program generally depends on more than one trait (Bernardo 2002; Lynch et al. 1998). Hence, determining which individuals to select to be the parents of the next generation forces the breeder to consider several different characteristics.

This is usually referred to as multiple-trait selection and implied selection for correlated traits. Not all the correlated traits are equally important or all independent of each other, but they are of interest for two main reasons in breeding programs.

Firstly, to understand the genetic causes of correlation through the pleiotropic action of genes or physical linkage of genes. Secondly, because it is key to understand how the improvement of one trait will trigger concurrent changes in other traits (Allard 1999).

There are many ways of selecting for multiple traits but these will not often be equally efficient. The most efficient method is that which results in the maximum genetic improvement per unit of time and effort expended (Hazel and Lush 1942; Smith 1936).

One might select each trait singly in successive generations (tandem selection) until each trait is improved to a desired level. Tandem selection is practical when some traits can be meaningfully evaluated in the earlier stages of a breeding program and other traits can be evaluated only later (Acquaah 2009; Burgess and West 1993; Hallauer and Miranda 1987). One might select for all the traits at the same time but independently, rejecting all individuals that fail to come up to a certain standard for each trait regardless of their values for any other of the traits (independent culling levels) (Hazel 1943). Only individuals that meet the minimum or maximum standards for each trait are selected.

Most breeders have to deal with selection of multiple traits simultaneously (in pure lines, inbred lines, hybrids, clones, and synthetics). Therefore, multi-trait selection using a selection index is an attractive approach (Hazel and Lush 1942; Hazel 1943; Williams 1962). In index selection, the component traits are combined into a score or index, in such a way that selection applied to the index, as if the index were a single trait, will yield the most rapid possible improvement of economic value.

If all traits were collected easily and at the same time, the index selection could be applied within phenotypic selection (PS). When some trait values are missing, GEBVs obtained by genomic prediction can be the basis for index calculations. A major challenge that remains in index selection is the apriori weight assignment of economic values for different traits. Each breeding program has potentials for genomic improvement in the traits of interests defined by the genetic composition of the breeding population which might make certain breeding goals more easily attainable for that breeding program compared to others. Pre-assigned economic weights do not necessarily represent specific potentials of a breeding program. A parent breeding population’s potentials can be defined as the lengths of all the paths between the mean BV of the individuals in this breeding population and the mean BV in a progeny breeding population that lead all the traits in the desired direction. If we assume that the gains in these traits have equal importance, then preferring longest path progeny population would mean we would be accumulating as many beneficial allele effects as possible in one cycle of breeding. The potentials concept can be extended to more than one generations, by calculating paths between the parent and progeny populations obtained in more than one cycle.

Index selection will not necessarily include the best individuals with respect to individual traits (for example see Supplementary Fig. 8. This can be seen as a drawback of the method because not including the best individuals for individual traits can lead to loss of beneficial alleles. In addition, index selection method does not control for inbreeding.

The multitrait breeding problem pose a fundamental question in terms of the best procedure to reach the breeding goals. In the last years, the great innovation in computer science have allowed to test new statistical methods to model uncertainty in multi-trait selection not just to improve selection (Bernardo and Yu 2007; Goddard 2009; Heffner et al. 2009; Meuwissen et al. 2001) but also as a tool to facilitate ideotype design in crop modelling (Casadebaig et al. 2016; Gouache et al. 2017; Martre et al. 2015; Picheny et al. 2017). Numerical models can predict the outcome of plant traits by simulating physiological processes and their interaction with the environment (Ghanem et al. 2015; Martre et al. 2015; Rötter et al. 2015).

In this article, we propose an approach to multi-trait breeding based on a multi-optimization framework by setting optimal compromise solutions (Pareto front) that should be identified by an effective and complete search procedure to let the breeder to carry out the best choice. A novelty of our approach is that it extends some of the previously proposed breeding approaches, such as optimal parental contributions to multiple traits. However, the MOOB framework provides a framework with which many other breeding problems can be answered. For instance, we have included an illustration of MOOB framework for selection of training populations in the discussions section.

This article describes methods for sustainable improvement of crops and animals in rapidly changing environments by modeling genetic variability, optimizing breeding choices that involve trade-offs across multiple-traits, while controlling for the negative impacts of excessively high selection intensities on useful genetic variability.

The aim objectives of this article are (i) introduce the multi-objective optimization framework for plant and animal breeding and (ii) to compare the efficiency of the methods of this framework with previous multiple trait approaches.

## Materials

### Wheat and barley datasets

The genetic material used in this study consists of two different datasets on wheat and barley. Both of these data sets were downloaded from the triticeae toolbox (https://triticeaetoolbox.org) and more details about these datasets are provided in Table 1. We will use a wheat and a barley data to illustrate the use of multi-objective optimized breeding (MOOB) approaches.

**Table 1:** Germplasm description summary and heritability values for each trait of Wheat and Barley datasets

	Wheat data	Barley
Individuals	250	300
Markers	22,620	4419
Environments	2	4
Traits	2	3
*Heritability*		
Yield	0.65	0.32
Grain protein content (GPC)	0.78	0.28
Height	–	0.44
*Genetic correlation*		
Yield–GPC	−0.3	−0.27
Yield–height	–	0.55
Height–GPC	–	−0.14

#### Model for estimating BVs

For each dataset, the GEBVs for the traits grain yield (GY) and grain protein content (GPC) were predicted from a multi-trait mixed model with environment as fixed effects and BVs of individuals as random effects having a zero centered matrix-variate normal distribution with a separable covariance structure (Akdemir and Gupta 2011; Henderson and Quaas 1976; Montesinos-López et al. 2016) for traits and genotypes (Supplementary Equation 1). A similar model was assumed for the random residual terms. The GEBVs obtained from the above model were centered and standardized to bring the GEBVs to the same scale.

### Simulations

The long-term performances of multi-trait breeding methods were evaluated by simulations. Beginning with two distinct founder genotypes, we have formed a population of *N* (*N* = 100, 200, 300, 400) genotypes with 1000 single nucleotide polymorphism (SNPs) at three chromosomes each; and carried this population through 100 generations of random mating. Two traits were defined simultaneously by attaching random quantitative trait loci (QTL) effects at 200 randomly selected loci on each chromosome where 100 of these were taken to have opposite sign effects for the different traits, using these effects to calculate the genotypic values for each individual and adding to each of these a value generated from a normal distribution with zero mean and variance equal to the variance of the genotypic values in the population. The traits in this initial population were negatively correlated and each had a heritability value of 0.5. Heritability of the traits were kept constant through the cycles of the simulations. The base population for breeding simulations were obtained by simulating 10 rounds of tandem selection over two traits with 50% selection intensity. Thirty replications of 16 cycles GS and 10 cycles of PS with tandem, index selection and culling, and 16 cycles with three multi-trait breeding methods recommended in the manuscript have been simulated starting from this base population. Marker effects were estimated from simulated phenotypic and genotypic data on the current population data at odd numbered breeding cycles.

## Methods

In this section, we will describe two new approaches along with the standard methods for multi-trait breeding. We will illustrate and compare these methods with empirical data sets and with simulation studies. More details about the multi-objective optimization techniques can be found in Coello (2006), Deb (2001), Konak et al. (2006) and references there in.

### Multi-objective optimization and related concepts

A single-objective optimization problem is defined as the minimization (or maximization) of a scalar objective function *f*(***x***) subject to inequality constraints *g*_*i*_(***x***) ≤ 0, *i* = 1, …, *m* and equality constraints *h*_*j*_(***x***) = 0, *j* = 1, …, *p*, where ***x*** is a *n*-dimensional decision variable vector.

Multi-objective problems are those problems where the goal is to optimize simultaneously *k* objective functions designated as: *f*_1_(***x***), *f*_2_(***x***), …, *f*_*k*_(***x***) and forming a vector function $F({\boldsymbol{x}}) = \left[ {f_\ell ({\boldsymbol{x}})} \right]_{\ell = 1}^k$ subject to inequality constraints *g*_*i*_(***x***) ≤ 0, *i* = 1, …, *m* and equality constraints *h*_*j*_(***x***) = 0, *j* = 1, …, *p*.

Although single-objective optimization problems may have a unique optimal solution, multi-objective optimization problems (as a rule) present a multiplicity of compromise solutions, i.e., Pareto optimal solutions are those solutions within the decision space whose corresponding variables cannot be all simultaneously improved.

Non-dominated ordering implements the concept of dominance (not to be confused with the dominance concept in genetics that describes the relationship between alleles of one gene) and classifies a population of solutions into boundaries according to their level of dominance. The first level includes all the non-dominated solutions, the second level are formed by the non-dominated solutions after excluding the solutions in the first level and this allocation process finishes when all solutions are allocated within their respective frontiers. After this process, the first-frontier solutions are not dominated by any other individual; however, they dominate the second frontier. Thus, solutions of the *i*th frontier dominate individuals of the (*i* + 1)th frontier, i.e, solutions can be sorted according to these frontiers (see Supplementary Fig. 1). Each circle in Fig. 2a, b is a genotype, and dominance ordering connects all genotypes of the same dominance with a line. The genotypes on the lower dominance ordering levels are preferable to genotypes in the higher ordering levels and these genotypes with lower dominance ordering levels should be assigned higher weights in selection.

Non-dominance ordering and assignment of parental contribution proportions based on the above ideas are demonstrated with an hypothetical example in Supplementary Fig. 1a–f.

#### Selecting a “good” solution on the frontier surface

At the end of a multi-objective optimization, the decision maker (DM) has to select the preferred solutions from the Pareto frontier; this can be a difficult task for high-dimensional multi-objective optimization problems. For this reason, decision-making support tools are developed to aid the DM in selecting the preferred solutions. The choice of a unique solution in the collection of Pareto optimal solutions depends on the knowledge of problem characteristics, and a solution in a particular model may not be the best in another model or environment. For two-dimensional and three-dimensional multi-objective optimization problems a strategy is to first plot the Pareto frontier followed by visual identification of the kink (knee) of the frontier as the region of preferred solutions. Some methods of finding knees in multi-objective optimization are described in Branke et al. (2004). An heuristic approach for identifying preferred solutions on the frontier can be defined by using the ideal solution concept and global criterion (see Supplementary Information). Some other decision support tools for multiple-criterion decision-making were described in Agrawal et al. (2005), Zio and Bazzo (2012), and Tušar and Filipič (2015).

#### Self-organizing maps (SOMs) for visualizing the Pareto optimal solutions

SOMs (Kohonen 1981, 1998) have been recommended for visualizing the Pareto optimal solutions for high-dimensional multi-objective problems (Obayashi and Sasaki 2003). Neural networks are used in learning tasks that are too complex for human brain to comprehend and SOM is a unsupervised neural networks technique for organizing complex or vast amounts of data by providing lower dimensional representations of data in manner that is most easily understood. Specifically, SOMs are a type of artificial neural network that provides a topology preserving mapping from the high-dimensional space to map units. The property of topology preserving means that the mapping preserves the relative distance between the points; points that are near each other in the input space are mapped to nearby map units in the SOM. The SOM can thus serve as a cluster analyzing tool of high-dimensional data and be used as a visual aid in determining a ‘good’ solution on the frontier surface. We have provided two examples that illustrate the use of SOMs in the context of MOOB in Supplementary Figs. 13 and 14.

### MOOB strategies

GS is being used increasingly in plant breeding to accelerate genetic gain (Crossa et al. 2010; Edriss et al. 2017; Gaynor et al. 2017; Roorkiwal et al. 2016). GS focuses on best performance of parents before mating, while genomic mating (GM) (Akdemir and Sánchez 2016) includes information on complementation of parents to be mated and thereby is more sustainable in the longer term.

The standard breeding approaches, such as PS, GS, GM, and pedigree-based prediction, can be used with any of these multi-trait breeding approaches.

In the remaining of this article, we assume that a high density marker data is available for the current breeding population from which the co-ancestry coefficients can be calculated, and that there is no pedigree information. The implementation of PS in our simulations did not use any genotypic information or pedigrees. Basically, it referred to selecting the individuals with best observed phenotypes to be parents in the next generation.

#### Non-dominated selection

One approach involves sorting the individuals in a breeding program according to non-dominance ordering using the (predicted) BVs over the traits of interest. The assignments of parental contributions are done by assigning higher weights to individuals at lower non-dominance order.

Non-dominance ordering and assignment of parental contribution proportions based on the above ideas are demonstrated with an hypothetical example in Supplementary Fig. 1a–f.

#### Multi-objective optimized genetic gains while controlling co-ancestry

##### Parental contributions in the context of MOOB

Let *A* be the additive genetic relationships between the individuals in the genetic pool (this matrix can be obtained from genome-wide markers for the individuals) and let ***c*** be the vector of proportional contributions of individuals to the next generation under a random mating scheme. The average inbreeding and co-ancestry for a choice of ***c*** can be defined as *r* = 1/2***c***′*A****c***. If ***b*** is the vector of GEBVs, i.e., the vector of BLUP estimated BVs of the candidates for selection, the expected gain is defined as *g*(***c***) = ***c***′***b***. Without loss of generality, assume that the breeders long-term goal is to increase the value of *g*(***c***).

Several authors (Brisbane and Gibson 1995; Clark et al. 2013; Meuwissen 1997; Sonesson et al. 2012) have proposed minimizing the average inbreeding and co-ancestry while restricting the genetic gain. These approaches find the parental proportions obtained by solving the following optimization problem: minimize *r*(***c***) = 1/2***c***′*A****c*** subject to ***c***′***b*** = *ρ*, and ***c***′1 = **1**′***c*** ≥ 0, where *ρ* is the desired level of gain. This problem is easily recognized as a quadratic optimization problem (QP). There are many efficient algorithms that solves QPs so there is in practice little difficulty in calculating the optimal solution for any particular data set. Recently, several allocation strategies were tested using QPs in Goddard (2009), Pryce et al. (2012), and Schierenbeck et al. (2011).

**Fig. 1:**
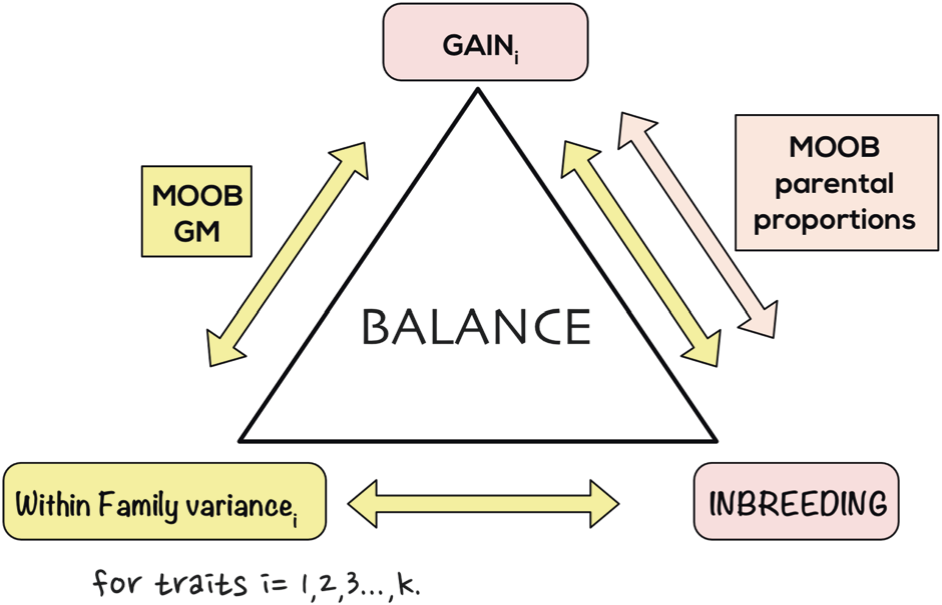
An abstraction of methods for improving multiple traits under parental proportions and genomic mating. MOOB multi-objective optimized breeding. Assigning parental contributions involve balancing gains for *k* traits and inbreeding. Genomic matings seeks to balance gains, within-family variances for *k* traits and inbreeding

We suggest the following extension of the above formulation for obtaining parental proportions in the multi-trait scenario: Let ***g***_1_, ***g***_2_, …, ***g***_*k*_ denote the *m*–dimensional vectors of GEBVs for *k* traits. We assume maximum is sought for each of these traits. As in Brisbane and Gibson (1995), Meuwissen (1997), Meuwissen et al. (2001), Wray and Goddard (1994) we want to keep inbreeding to minimum. This defines the multi-objective optimization problem (see Fig. 1); more formally we are looking to solve the maximization of the vector function

1 $$F({\boldsymbol{c}}) = \left[ {f_\ell ({\boldsymbol{c}})} \right]_{\ell = 1}^{k + 1},$$

with $f_1({\boldsymbol{c}}) = {\boldsymbol{c}}\prime {\boldsymbol{g}}_1$, $f_2({\boldsymbol{c}}) = {\boldsymbol{c}}\prime {\boldsymbol{g}}_2, \ldots$, $f_k({\boldsymbol{c}}) = {\boldsymbol{c}}\prime {\boldsymbol{g}}_k$, $f_{k + 1}({\boldsymbol{c}}) = - {\textstyle{1 \over 2}}{\boldsymbol{c}}\prime A{\boldsymbol{c}}$ subject to inequality constraints *c*_*i*_ ≥ 0, *i* = 1, …, *m* and equality constraint $\mathop {\sum}\nolimits_{i = 1}^m {\kern 1pt} c_i - 1 = 0$.

A slight modification of the above aims to penalize negative genetic correlations between trait pairs, and it involves changing $f_{k + 1}({\boldsymbol{c}}) = - {\textstyle{1 \over 2}}{\boldsymbol{c}}\prime A{\boldsymbol{c}}$ to

2 $$f_{k + 1}({\boldsymbol{c}}) = - \frac{1}{2}{\boldsymbol{c}}\prime \left( {\left( {k - 2\mathop {\sum}\limits_{i = 1}^{k - 1} {\kern 1pt} \mathop {\sum}\limits_{j > j}^k {\kern 1pt} {\mathrm{\Psi }}_{i,j}} \right)A} \right){\boldsymbol{c}},$$

where Ψ_*i*,*j*_ denotes the genetic correlation between traits *i* and *j*. Both Eqs. (1) and (2) can be solved using QP.

The second class of multi-trait breeding schemes assigns parental proportions using one of the preferred solutions on the Pareto surface for the multi-objective problems stated in Eqs. (1) or (2).

#### GM in the context of MOOB

As opposed to the continuous parentage contribution proportions solutions in the GS method, the GM method gives the list of parent mates of the progeny (Akdemir and Sánchez 2016; Gorjanc and Hickey 2018; Kinghorn and Shepherd 1999; Kinghorn 2011). Multi-objective optimization problem (assuming maximization is sought for the trait) of the GM problem involves minimization of −*Gain*(*P*), −Cross_Variance(*P*) and *Inbreeding*(*P*) with respect to mating plan *P*. Here, *Gain*(*P*) represents the expected BVs, −Cross_Variance(*P*) represents the expected variance in the BVs and *Inbreeding*(*P*) measures the expected inbreeding of progenies obtained according to mating plan *P*.

Note that the measures of inbreeding in the optimal parental proportions and the GM approaches are different: The former is a measure of group co-ancestry while the latter is a measure of inbreeding of specific matings.

The expected gain for a mating plan can be calculated from the mid-parent genetic values. There are several alternative measures of inbreeding based on mating plans (Leutenegger et al. 2003; Wang 2011). In Akdemir and Sánchez (2016), we have used a measure derived under the infinitesimal genetic effects assumption proposed by Quaas (1988) and Legarra et al. (2009). Measures of expected cross-variance (related to the risk measure in Akdemir and Sánchez (2016) and also to the usefulness concept in Jannink (2010)) can be obtained using the results in Akdemir and Sánchez (2016) under the assumption of unlinked markers. An alternative approach would be to use simulated progenies to calculate the cross-variances. One can also include information about the LD in these simulations. For example, Bernardo (2014) and Mohammadi et al. (2015), suggested to simulate progenies using the parental genotypes and a genetic map. Other measures of cross-variance were proposed in Lehermeier et al. (2017) and Zhong and Jannink (2007).

Extension to multi-trait GM for a *k* trait problem (assuming maximization is sought for traits) is defined by the optimization problem which seeks minimization of −*Gain*(*P*)_*i*_, −Cross_Variance(*P*)_*i*_ for *i* = 1:,2, …, *k* and *Inbreeding*(*P*) with respect to mating plan *P* (see Fig. 1).

Also note that the Results section below includes results obtained using the multi-objective optimized parental proportions approaches. The only instance of an example of the mating-based approach is reserved to the supplementary (Supplementary Figs. 13 and 14), where we display the frontier solutions for the GM-based approach. The readers can refer to the related R package “GenomicMating v2.0” (Akdemir et al. 2018) for an implementation of the multi-objective optimized GM designs.

## Results

### Wheat and barley data sets

**Fig. 2:**
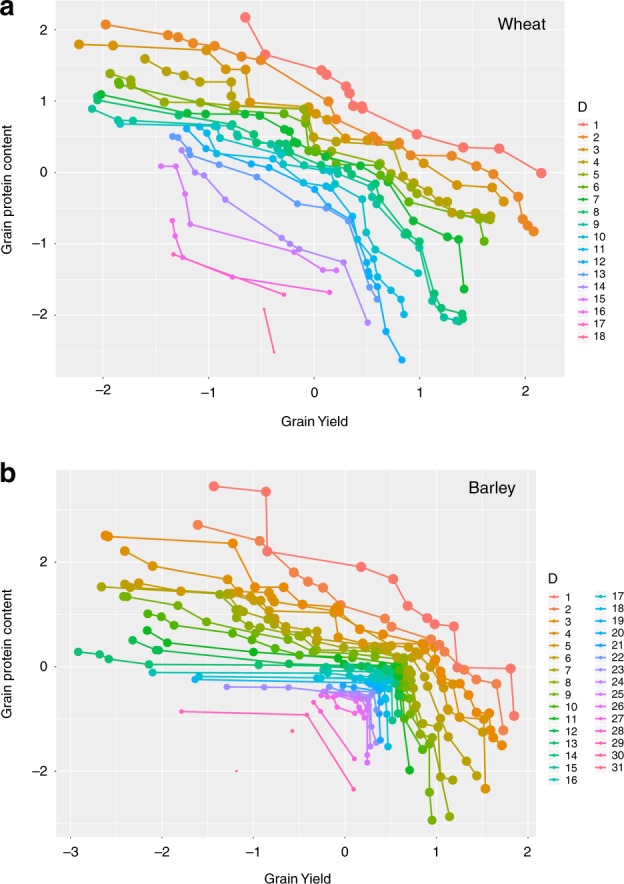
These figures are obtained by plotting GEBVs from model in (eq:model) for grain yield and grain protein content in wheat (**a**) and barley (**b**) dataset. Each circle represents a genotype, and dominance ordering connects all genotypes of the same dominance with a line. The genotypes on the lower dominance ordering levels are preferable to genotypes in the higher ordering levels. Genotypes with lower dominance ordering levels should be assigned higher weights in selection. There are 18 and 31 levels of dominance for wheat and barley data sets. The axes in these figures measure the standardized GEBV values (i.e., centered by mean, scaled by standard deviation) for grain yield and grain protein content

Figure 2 and Supplementary Fig. 2 show the non-dominated orderings for wheat and barley datasets based on the GEBVs of traits GY and GPC.

**Fig. 3:**
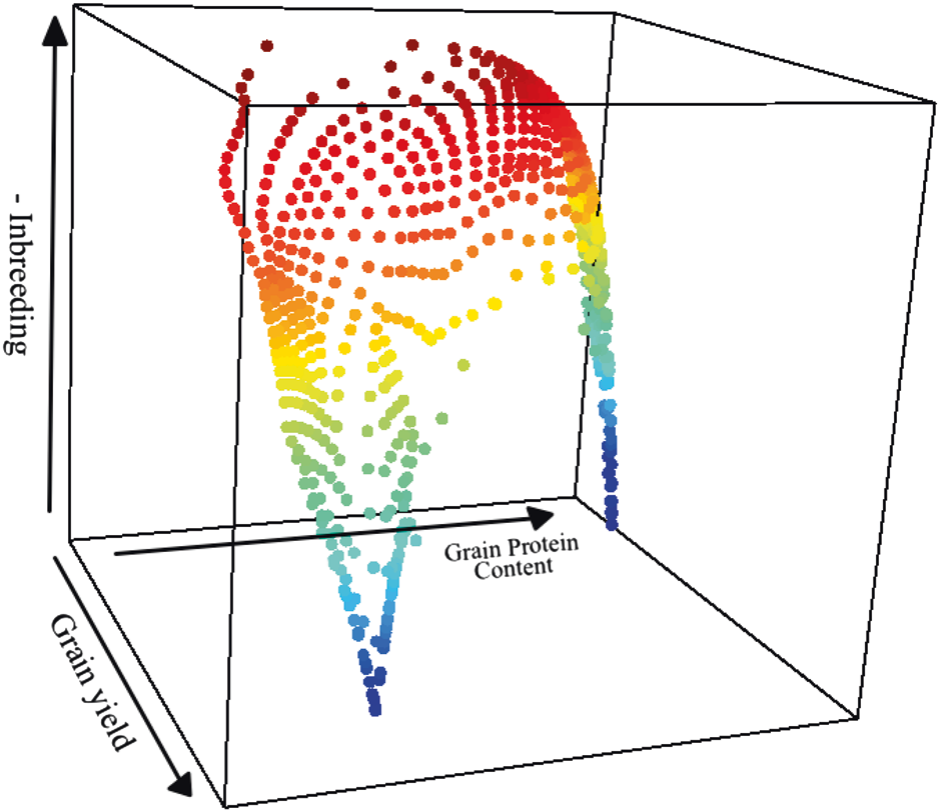
Pareto optimal solutions for parental contributions (wheat data) obtained by solving the optimization problem giving in Eq. (1) for improving grain yield (GY) and grain protein content (GPC) while controlling group coancestry, i.e, we assume we want to maximize GY, GPC, and the negative of inbreeding. The redness of the points indicates closeness to ideal solutions as calculated by the formula in Supplementary Information Equation (Eq. (2))

The frontier surface related to the optimal parental proportions for the wheat and barley datasets is given in Fig. 3 and Supplementary Fig. 3. These figures show the frontier surfaces obtained by plotting Pareto optimal solutions for parental contributions obtained by solving the optimization problem given in Eq. (1) for improving GY and GPC while controlling group coancestry. A good solution can be visually detected by closely examining this surface to find a acceptable kink point, i.e., solutions of the Pareto-optimal front where a small improvement in one objective would lead to a large deterioration in at least one other objective. These solutions are sometimes also called “knees”.

**Fig. 4:**
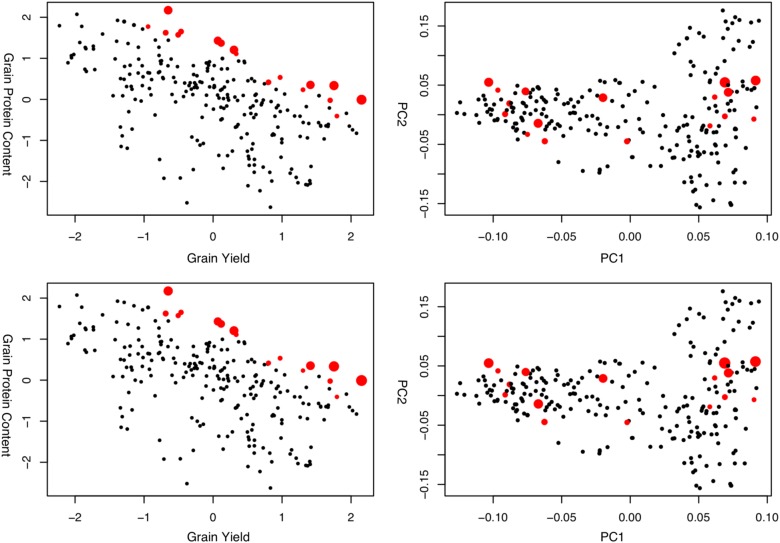
Two ‘good’ solutions on the wheat frontier curve obtained from Fig. 3 for the wheat data. Red points indicated the individuals that have non-zero parental proportions. The size of the points are proportional to the magnitude of the parental contributions. The figures on the right side, represent the same information but on the first two principal components of the genotyping marker space. PC principal components

Figure 4 shows an example of two “good” solutions on the wheat frontier curve obtained from Fig. 3. Supplementary Fig. 4 as calculated by Eq. (2) also shows “good” solutions for the barley dataset. Note that non-dominated ordering-based approaches (Supplementary Fig. 2) give similar solutions to optimized parental proportions approach. Nevertheless, solutions for the parental proportions represent the control in inbreeding and have different weights for the individuals on the same level of dominance.

The multi-trait breeding described in this manuscript can also be used to improve one or more traits in several target environments. For example, a common breeding goal is improvement of yield in multiple environments. The non-dominance ordering for the GEBVs for GY in the four barley dataset environments (dry–irrigated × high–low nitrogen) are given in Supplementary Fig. 7.

### Long-term performance evaluated by simulations

**Fig. 5:**
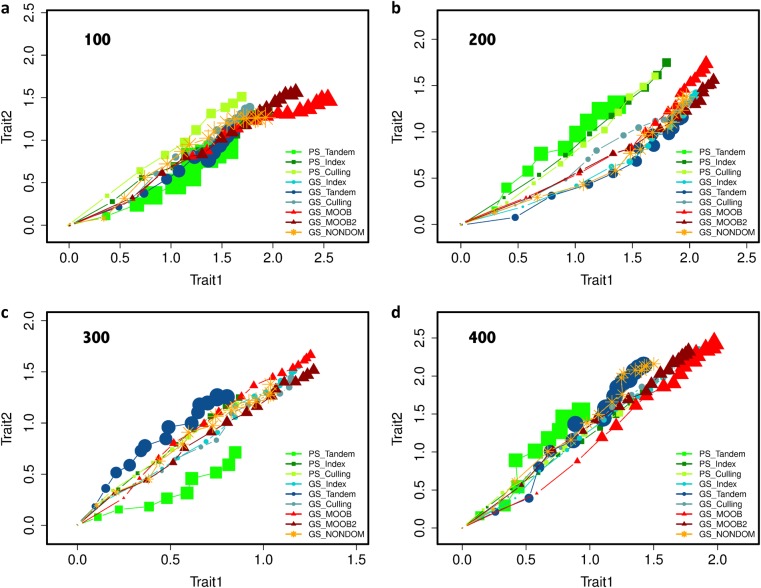
Simulations: the results from 30 replications of 16 rounds GS and 10 rounds PS with tandem, index selection (equal weights for traits) and independent culling, and 16 rounds with three multi-trait breeding methods. Breeding population sizes 100, 200, 300, and 400. Data points (in the trend lines) represent the improvement in Trait 1 and Trait 2 in consecutive breeding cycles. The changing sizes of points represents the variability of different breeding methods at each cycle over replicated trials of the experiment (30 reps) starting from the same initial population, the larger points corresponding to larger variances (the total variance of the mean breeding values obtained by a breeding method at a certain cycle calculated over the replications)

Figure 5a–d shows the results from simulations for the study of the long-term behavior of PS and GS. Here, we use the standard methods of index and tandem selection, as well as, the independent culling method, along with the results for non-dominated selection, and the two forms of MOOB schemes. The index weights for both traits were set to 0.5 in the simulations.

For the methods which involve genomic prediction, a multivariate mixed model was used to predict GEBVs of the traits at odd numbered cycles using the phenotypic values in that cycle as training data, which was then used in the next even numbered cycle to predict GEBVs of individuals in this cycle. The estimates of genetic covariance parameters were updated through the odd numbered cycles using the fitted model.

The simulation results indicate that the MOOB approach is more efficient (resulted in higher values in both Trait 1 and Trait 2 at the end of the breeding cycles) than classical methods. For all the population sizes, the MOOB approach performed more efficiently than any other methods by attaining the highest values of the BVs for both traits at the end of breeding cycles. Compared to the best alternative breeding strategy, the gains from multi-objective optimized parental proportions approaches were about 20–30% higher at the end of long-term simulations of breeding cycles.

Selection of solutions on the frontier during these simulations were done using a weighted distance to the ideal solution (which was taken as the optimal values for the three statistics within the solutions on the frontier). The weights for populations of size 100 and 200 were fixed at 0.95, 0.025, and 0.025 corresponding to the measures of inbreeding, gain in Trait 1 and gain in Trait 2. We have increased the intensity of selection for populations of size 300 and 400 by changing these weights to 0.9, 0.05, and 0.05. Note that, these values will be population specific and were fixed here only for the purposes of computer simulation of many cycles of breeding as described above. Decision support tools described previously should be utilized when applying the MOOB methods.

## Discussion

A major task of breeders is to increase the frequency of favorable alleles of quantitative traits, controlled by a large number of genes. After the choice of germplasm, a breeder uses some type of cyclical selection program to maximize the genetic improvement of desired traits. The important aspect is that superior materials selected for the breeding population should be recombined to obtain a new breeding population. Therefore, it is important to incorporate recurrent selection into classical breeding programs (Hallauer and Carena Filho 2010). One of the drawbacks of incorporating GS in breeding programs or even the traditional pedigree-based selection could potentially lead to greater rates of inbreeding than PS, especially when the accuracy of the method is low to moderate (Lin et al. 2017). Although, it has been shown that the inbreeding rate per generation of GS is less than pedigree selection (Daetwyler et al. 2007), GS could lead to higher inbreeding rates per year when compared to PS (Lin et al. 2017). A potential consequence of higher inbreeding is decreased survival, growth, and reproduction in outbreed plants (Lin et al. 2017). In addition, the response to selection per er cycle might be drastically reduced. Therefore, the accumulation of inbreeding from GS should be controlled to avoid those detrimental effects.

In this study, we have showed that optimization strategies that controls the loss of genetic diversity (MOOB framework) allows to (i) better conservate genetic variance in the breeding population, (ii) to better estimate of GEBVs and (iii) to have minor repercussions on the genetic gain (Eynard et al. 2017; Gorjanc et al. 2017).

In addition, we believe that the main promise of MOOB is that they provide the breeders with decision support tools that allow optimal exploitation of the breeding material. Breeders are routinely faced with the challenge to obtain an optimal decision with respect to multiple criteria. There is a clear need for planning tools to support effective decision making in this domain assisting the DMs in choosing an adequate strategy within the possibilities offered by the decision space.

Breeding through selection of superior genotypes for use as parents in future generations usually involves selection, which are based on more than one trait (Johnson et al. 1988). For example, although the GY is usually the primary trait of interest for most crops; plant height, earliness, stability, grain quality, stalk quality, abiotic and biotic stress tolerance, responsiveness, etc. (Mendonça et al. 2017, 2016) are also economically important traits. In assessing grain quality, elements of human subjectivity can be reduced by using multiple clearly defined traits observed by multiple raters to model grain quality (Stansell et al. 2017). Thus, simultaneous selection for several traits is necessary if recurrent selection methods are used because the selection that emphasizes only one trait can be detrimental to the overall agronomic performance of the germplasm (Hallauer and Carena Filho 2010). Consequently, the aim is to combine the favorable alleles that are present in different individuals, forming new and superior haplotypes.

As contrasted to the index selection methods, which require a prior decision on economic weights, the MOOB methodology involves selection of a ‘good’ solution among the optimal compromise solutions with the aid of decision support tools. The DM can come to a final decision by examining the frontier surface, or by using the ideal solution concept, by using the visualizations using SOMs. This addresses a major challenge in the application of index selection for many breeding programs: “how to assign weights?”

In our simulations, we have seen that MOOB outperforms other breeding approaches including the index selection. We would like to note that none of the compared approaches except MOOB controls for loss of genetic variation this is the main advantage of MOOB compared to the other methods. Nevertheless, it is possible also to imagine a scenario where the gains of and index is balanced with loss of genetic variation. This will lead to a muti-objective optimization problem which it is solved under MOOB framework.

We note that the multi-objective optimization methods can also be used in selection of training populations for genomic prediction. There has been many approaches that used a single selection criteria for designing genomic prediction training populations with the promise of improving genomic prediction accuracies and therefore improving expected gains from GS. Most of the literature is devoted to demonstrating the advantages and disadvantages of using one of these methods over the other (Akdemir et al. 2015; Isidro et al. 2015; Rincent et al. 2012). This debate can be somewhat circumvented by designing training populations that are optimal for multiple design criteria at the same time, in the multi-objective optimization framework. For example, a multi-objective optimized training population selection approach might seek solutions that balances genomic diversity in the training population, genetic closeness to a target population (the GS model trained in the training population will be used to predict GEBVs in the target population) in addition to some other criteria related to selection of training populations. This point is illustrated in Supplementary Fig. 15.

In this manuscript, we have used the multi-objective optimization approach in the context of multi-trait selection in breeding to efficiently select multiple traits simultaneously. Breeding for multiple traits is a real problem faced by breeders, usually because the choice of weightings in a selection index is inherently difficult due to the number of contributing factors. This paper presents a potential solution to this problem. In addition, MOOB is a flexible framework that can be used to answer many other problems in breeding, such as design of training populations. Future research directions therefore include finding other applications for MOOB related to breeding and more thoroughly evaluating the promises of this new framework in breeding.

## Electronic supplementary material

MOOB-SI_short.pdf
