# Supplementary material for: Multi-objective optimized genomic breeding strategies for sustainable food improvement
Source: Heredity (Edinb). 2018 Sep 27;122(5):672–83. doi: 10.1038/s41437-018-0147-1 (PMC6461918; doi:10.1038/s41437-018-0147-1)
Supplement: Supplementary file 1 — MOOB-SI_short.pdf [file 41437_2018_147_MOESM1_ESM.pdf]

Supplementary Information: Multi-objective optimized genomic breeding strategies for sustainable food improvement

Deniz Akdemir[a,**], William Beavis[b], Roberto Fritsche-Neto[c], Asheesh K.Singh[b], and Julio Isidro-Sánchez[d]

[a]Cornell Statistical Consulting Unit, Cornell University, Ithaca, NY, USA

[b]Department of Agronomy, Iowa State University, Ames, Iowa, USA.

[c]Department of Genetics, “Luiz de Queiroz” Agriculture College, University of Sao Paulo, Brazil

[b]Department of Agronomy, Iowa State University, Ames, Iowa,

USA

[c]Agriculture & Food Science, Animal and Crop section,

University College Dublin,Dublin, Ireland

[**]Correspondance: E-mail: akdemir@cornell.edu

August 9, 2018

Methods

Model for estimating breeding values

More specifically, the model that is used is given by

$$Y = XB + ZG + E, \tag{1}$$

where Y is the $n \times d$ response variable, X is the $n \times q$ design matrix of $q \times d$ the fixed effects B, Z is a $n \times q$ design matrix of the $q \times d$ random effects G, and E is the $n \times d$ matrix of residual effects. The random effects and the residual are independently distributed, and have matrix variate distributions ($G \sim N_{q\times d}(0_{q\times d}, K, \Sigma)$and $E \sim N_{n\times d}(0_{n\times d}, R, \Sigma_E)$) where K is a $q \times q$ relationship matrix, Σ is a $d \times d$ covariance matrix, R is a $n \times n$ covariance matrix, Σ_E is a

$d \times d$ covariance matrix. An early reference to this muti-trait model appears in [4].

Multi-objective optimization concepts

A vector $u = (u_1, \ldots, u_k)$ is said to dominate another vector $v = (v_1, \ldots, v_k)$ (written as $u \succeq v$) if and only if u is partially less than v ,i.e., $\forall i \in 1, \ldots, k$, $u_i \leq v_i \wedge \exists i \in 1, \ldots, k\ u_i < v_i$. Pareto optimal solutions are those which, when evaluated, produce vectors whose performance f_i cannot be improved without adversely affecting another f_j, $i \neq j$. In a minimization problem, a solution $\boldsymbol{x}$ is said to be Pareto optimal if and only if there is no $\boldsymbol{x}'$ for which $F(\boldsymbol{x}')$ dominates $F(\boldsymbol{x})$, i.e., there exists no feasible vector $\boldsymbol{x}'$ which would decrease some criterion without causing a simultaneous increase in at least one other criterion.

For a multi-objective problem, $F(\boldsymbol{x})$, the Pareto Optimal Set, P^*, is defined as: $P^* := \{\boldsymbol{x} : \neg\exists \boldsymbol{x}' F(\boldsymbol{x}') \succeq F(\boldsymbol{x})\}$.

The Pareto front PF^* is defined as: $PF^* := \{u = F(\boldsymbol{x}) | \boldsymbol{x} \in P^*\}$.

Non-dominance ordering and assignment of parental contribution proportions based on the above ideas are demonstrated with an hypothetical example in Figure 1. (a-f). In this figure, genotype L1 is nondominated

(so as L12, L15, L9, L11) with respect to GY and GPC since there are no other genotypes in this set when replaced with L1 that would not involve decrease in at least one of BVs. For example, replacing L1 with L11 gives an increase in GY value but a decrease in the GPC (red arrow). Replacing L1 with L9 gives an increase in GPC but a decrease in the GY (black arrow). Genotype L4 is dominated by L1, because by replacing it with L1 we can increase both BVs. The genotypes in dominance Level 1 are the set of non-dominated individuals. The genotypes in dominance Level 2 (red points) are obtained as the non-dominated genotypes in the smaller subset obtained by removing the genotypes in Level 1. This process is continued until all the genotypes are assigned to their dominance levels. The gray star refers to an example of an ideal genotype with respect to the set of genotypes. Weights can be assigned inversely proportional to the distance from ideal solution. (b) Parental proportions are inversely proportional to dominance levels. (c) Parental proportions are inversely proportional to squared dominance levels. (d) Parental proportions are obtained with respect to the non-dominance counts over combinations of traits. L12 is non dominated with respect to Grain protein and also when we consider the two traits at the same time, so gets a weight proportional to 2. L1 is only non-dominated with respect to

two traits at a time, so gets a weight proportional to 1. (e) Is obtained by taking the mean of the proportions in (c) and (d). (e) Parental proportions inversely proportional to the distances obtained using formula 2 using the ideal solution, $p = 2$ and equal weights for traits. Bar-plots are colored with respect to dominance levels.

Figure 1: (a) This is a scatterplot for breeding values (BVs) of grain yield (GY) and grain protein content (GPC) for 15 genotypes.

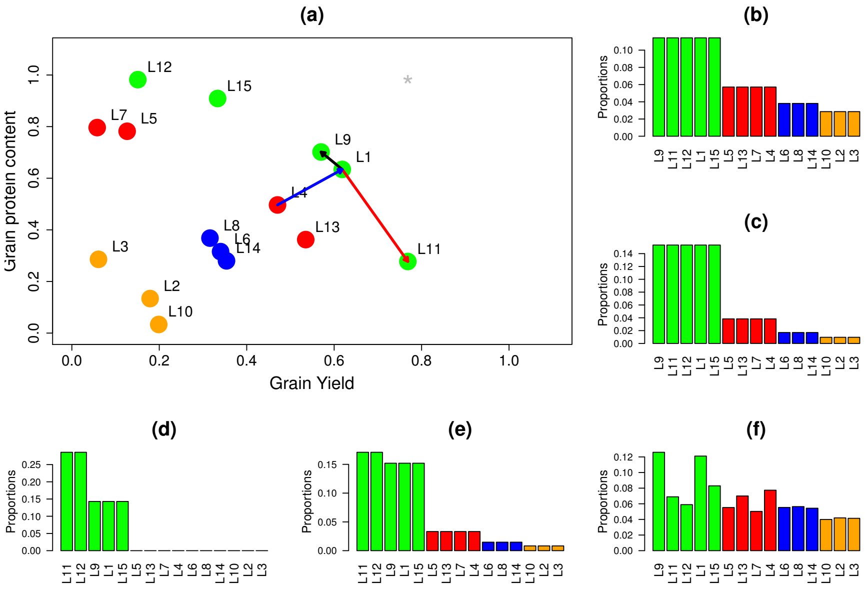

Multi-objective optimization techniques

In general, it is not possible to find an analytic expression of the line or surface that contains the Pareto optimal solutions. Several techniques are used to find representative points on the Pareto frontier:

Scalarization (Linear combination)

If a solution $\boldsymbol{x}$ to the general multi-objective problem is non inferior, then there exist $w_l = 0$, $l = 1, 2, \ldots, k$ (w_r is strictly positive for some $r = 1, 2, \ldots, k$), and $\lambda_i = 0$, $i = 1, 2, \ldots, m$, such that:

$$\sum_{l=1}^{k} w_l l \delta f_l(\boldsymbol{x}) - \sum_{i=1}^{m} \lambda_i \delta g_i(\boldsymbol{x}) = 0.$$

This condition is named Kuhn-Tucker Condition (KTC) and is necessary for a non-inferior solution. When all of the $f_l(\boldsymbol{x})$ are concave and $\boldsymbol{x}$ belongs to a convex set, they are sufficient as well. Since KTC is sufficient for non-inferiority, non-inferior solutions might be found by solving a scalar optimization problem in which the objective function is a weighted sum of the components of the original vector-valued function. That is, the solution to the problem: $\sum_{i=1}^{k} w_i f(\boldsymbol{x})$, where $w_i \geq 0$ for all i and strictly positive for at least one objective, is usually non inferior. Then on inferior set and the set of non-inferior solutions can be generated by varying the weights w_i in

the objective function.

The reduction of the problem to a single-objective function means to make all alternatives comparable with a preference framework that becomes a total order. Hence w_i values choice is very important to achieve the final decision and, for this reason, value choice is made by the decision maker. However, the decision maker, in order to choose the coefficients, must have a clear perception of how this choice effects all the functions with respect to each other.

The main advantages of this method are its simplicity (in implementation and use) and its efficiency (computationally speaking). Its main disadvantage is the difficulty to determine the appropriate weight coefficients to be used when enough information about the problem is not available (this is an important concern, particularly in real-world applications). Also, a proper scaling of the objectives requires a considerable amount of extra knowledge about the problem. To obtain this information could be a very expensive process. A more serious drawback of this approach, is that it cannot be used to generate certain portions of the Pareto front when the conditions of KTC are not satisfied, regardless of the weights combination used. Nevertheless, aggregating functions could be very useful to get a preliminary sketch of

the Pareto front of a certain problem or to provide prior information to be exploited by another approach.

Other scalarization methods include L_p-norm, Chebyshev and the single-objective product formulation. For each of these scalarizations, a characterization of the Pareto set can be obtained by varying the scalarization parameters and solving many single-objective optimization problems. Ideally, the points returned by the scalarized problems should be sufficiently spread out in the efficient frontier.

The ϵ-constraint method

Besides the scalarization by linear combination approach, the ϵ-constraint method is probably the best known technique to solve multi-objective optimization problems. There is no aggregation of criteria, instead only one of the original objectives is minimized while the others are transformed to constraints. The idea was introduced by [2]. Through this approach among p objective function only one is kept as such, the other $p-1$ are transformed in constraints fixing threshold values ϵ_k (with $k = 1, \ldots, p, k \neq j$) over them (if functions must be minimized). Therefore, the problem: $minF(\boldsymbol{x})$ is substituted by the ϵ-constraint problem: $minf_j(\boldsymbol{x})$ $f_k(\boldsymbol{x}) \leq \epsilon_k$, $k = 1, \ldots, p$, $k \neq j$. The main disadvantage of this approach is its (potentially high) computa-

tional cost, also due to the preliminary individuation of ϵ_i values.

Finding non-dominated solutions

Several algorithms exist for finding the non-dominated set in from a larger set. Popular Kung algorithm ([5]) involves first sorting the population in descending order in accordance to first objective function. Afterwards, the population is recursively partitioned as top (T) and bottom (B) sub-populations. As top half (T) is better in objective in comparison to bottom half (B) in first objective, so we check the bottom half for domination with top half. The solution of B which are not dominated by solutions of T are merged with members of T to form merged population M. Another algorithm is the Jun Du Algorithm ([3]).

Selecting a "good" solution on the frontier surface

Ideal solution concept and global criterion:

Let $\boldsymbol{x}_i^*$ be a vector which optimizes the ith objective function $f_i(\boldsymbol{x})$ for $i = 1, 2, \ldots, k$. Then the vector $[f(\boldsymbol{x}_1^*),\ f(\boldsymbol{x}_2^*),\ \ldots,\ f(\boldsymbol{x}_k^*)]'$ is ideal for an multi-objective problem and is consequently called the ideal vector.

The global criterion method aims to minimize a function (global criterion) which is a measure of how close the DM can get to the ideal vector. A measure

of closeness to the ideal solution is a family of L_p-metrics defined as follows:

$$L_p(F) = \left[\sum_{l=1}^{k+1} \left| \frac{f(\boldsymbol{x}_1^*) - f(\boldsymbol{x})}{f(\boldsymbol{x}_1^*)} \right|^p \right]^{1/p} .$$

If the functions values are normalized to the range $[0, 1]$, then the above formula becomes

$$L_p(F) = \left[\sum_{l=1}^{k+1} |1 - f(\boldsymbol{x})|^p \right]^{1/p} .$$

Finally, if weights are attached to the functions $f_1(\boldsymbol{x}), f_1(\boldsymbol{x}), \ldots, f_{k+1}(\boldsymbol{x})$ a weighted version can be written as

$$L_p^{\boldsymbol{w}}(F) = \left[\sum_{l=1}^{k+1} w_l \, |1 - f(\boldsymbol{x})|^p \right]^{1/p} . \tag{2}$$

In the remaining of this manuscript, we have used $p = 2$ which coincides with the Euclidean distance.

Ideal Solution for GEBVs

A simple estimator, say $maxf_i$, for the ideal solution $f(\boldsymbol{x}_i^*)$, for trait i in a certain breeding population is the maximum observed value for that trait. It is also possible to estimate this quantity by calculating the maximal estimated genomic value using the marker effects estimates. We use the former approach in the remaining of this article. When calculating the distance from the ideal solution, the objective function values were scaled to the range $[0, 1]$

using the transformation

$$f^*(\boldsymbol{x}) = \frac{f_i(\boldsymbol{x}) - minf_i}{maxf_i - minf_i},$$

for trait i; and $minf_i$ is the estimate for the worst value of the trait i, it is calculated in the same fashion as $maxf_i$.

Other multi-trait breeding approaches

Supplementary Figure 5 displays the parental contribution proportions obtained by non-dominance counts for 100 lines with the highest proportions for the improving yield and protein in the four environments.

Supplementary Figures 8 and 9 display the individuals that would be identified by the classical multi-trait breeding schemes culling, tandem and index selection for the wheat and barley datasets. These can be contrasted with the Figures 2 a and b and also with the Figure 4 and Supplementary Figure 4. Note that among the classical methods index selection will give the closest results to the multi-objective optimized breeding methods.

Multi-objective training population design

Two examples with different sets of selection of training populations related optimality criteria where we display Pareto fronts for training populations of

Figure 2: This figure represents the non-dominance ordering of the individuals in the barley dataset for three traits. Barley data: Dominance ordering based on three traits, 13 levels of dominance. The GEBVs for height, grain yield and protein from barley data are plotted with the dominance ordering of these individuals indicated by the lines of different color.

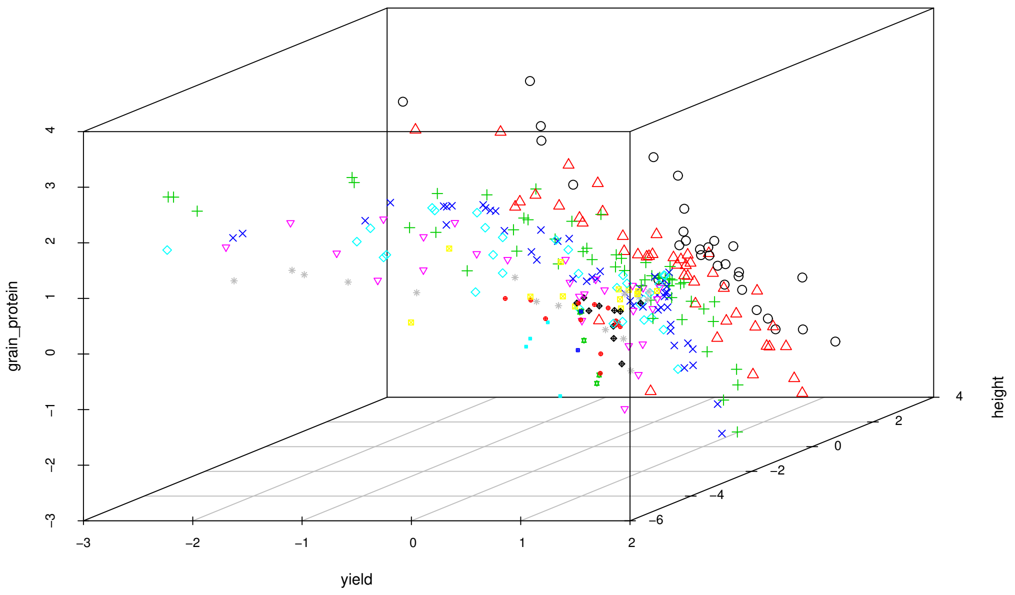

Figure 3: Pareto optimal solutions for parental contributions (Barley data) obtained by solving the optimization problem giving in Equation (1) for improving grain yield (GY) and grain protein content (GPC) while controlling coancestry, i.e, we assume we want to maximize GY, GPC and the negative of inbreeding. The redness of the points indicates closeness to ideal solutions as calculated by the formula in Supplementary Equation (2).

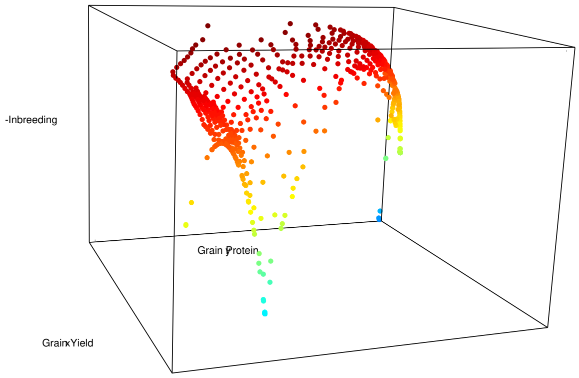

size 100 (selected from the remaining genotypes in the wheat dataset after reserving a random sample of genotypes as a target population). Some examples of optimality criteria include determinant optimality criterion (minimizes the determinant of the covariance matrix of the model coefficient estimates

Figure 4: Three 'good' solutions on the barley frontier curve obtained from Supplementary Figure 3. Red points indicated the individuals that have non-zero parental proportions. The sizes of the points are proportional to the magnitude of the parental contributions. The figures on the right side, represent the same information but on the first two principal components of the genotyping marker space.)

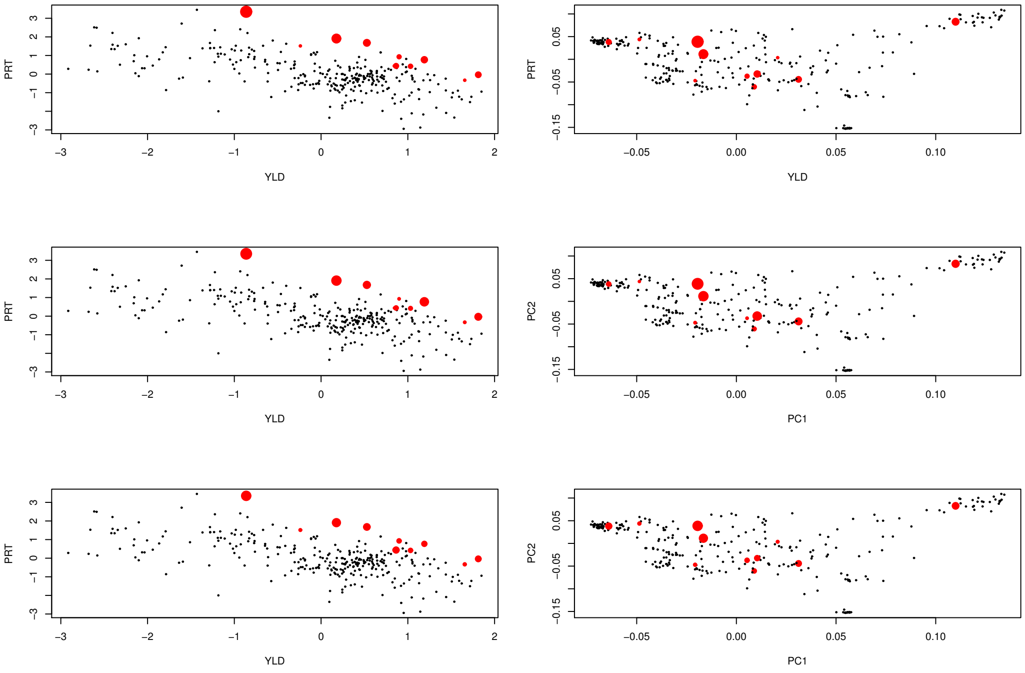

Figure 5: Barley data: parental contribution proportions obtained by non-dominance counts for 100 lines with the highest proportions for the improving yield in four environments.

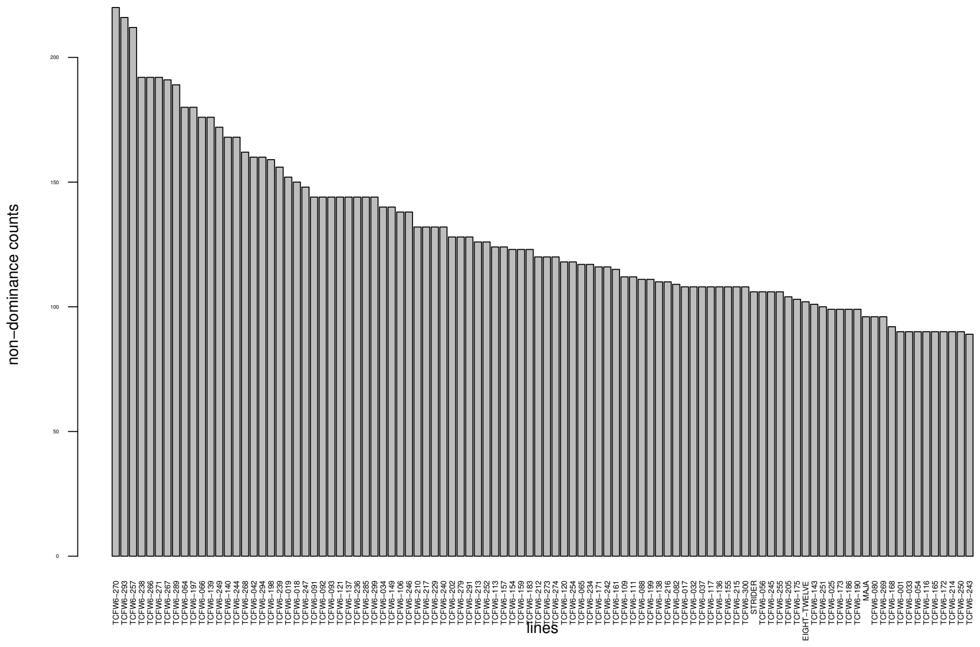

Figure 6: Barley data: The dimensions correspond to negative of inbreeding, and average gains based on GEBVs for grain yield (GY), height and grain protein content (GPC). Each point on the 3 dimensional scatterplots correspond to a Pareto optimal solution for parental contributions. Blueness of the points measure the closeness to the ideal solution as calculated by Equation (2).

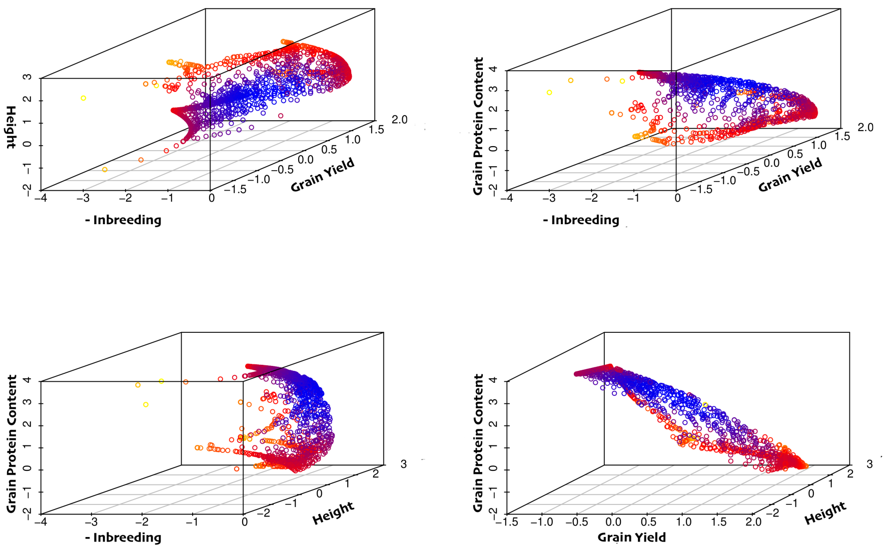

for a principal components regression model based on the markers; genomic distance based criteria such as mean or the minimum distances among genotypes in the training set, mean or maximum distance to the target set of genotypes. In the examples below, determinant optimality criterion was

Figure 7: Barley data: Dominance ordering based on yield in 4 environments (dry-irrigated × high-low nitrogen). The environment specific GEBVs for grain yield for barley data are plotted with the dominance ordering of these individuals indicated by the lines of different color.

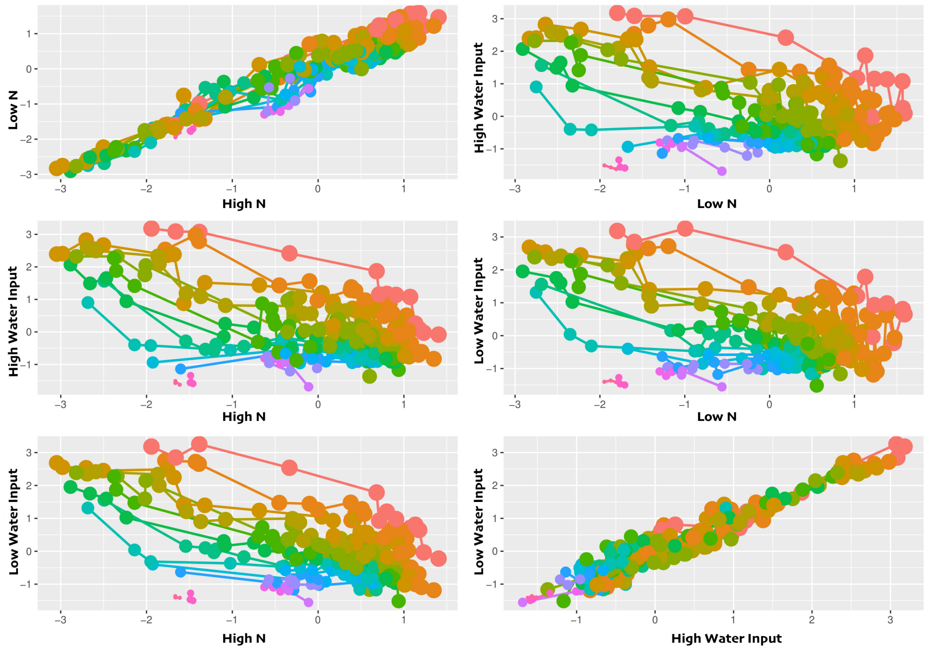

calculated using the first 50 principal components of the marker matrix as suggested in the R package STPGA [1] and the distance based criteria were calculated using the Euclidean distance matrix calculated using the marker matrix. The multi-objective optimization problem is setup such that we are seeking solutions (lists of training sets of size 100) to minimize these different criteria.

Figure 8: Wheat data: Other multi-trait breeding approaches

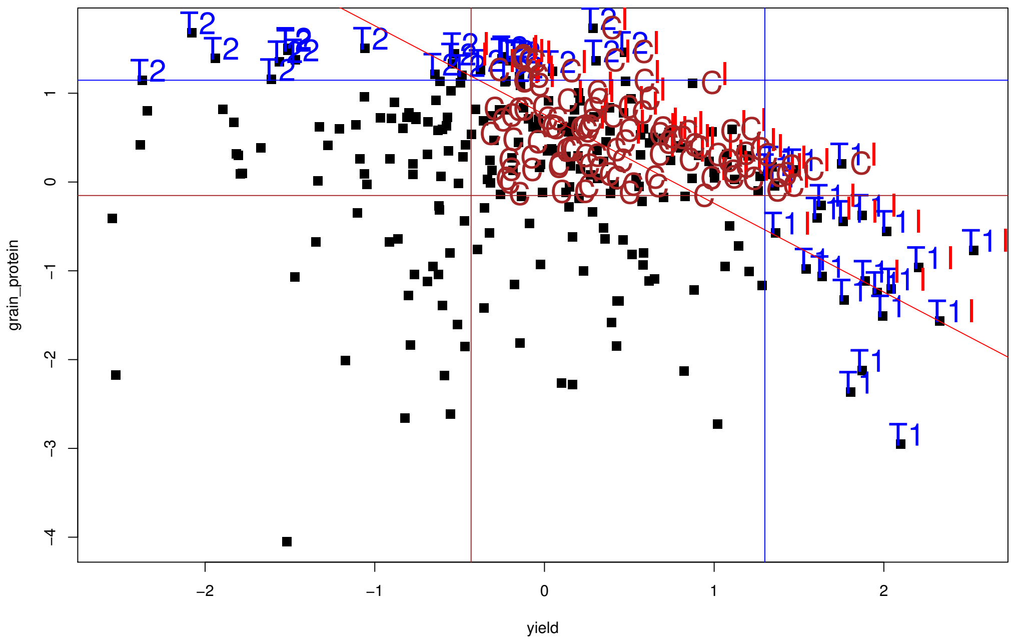

Figure 9: Barley data: Other multi-trait breeding approaches

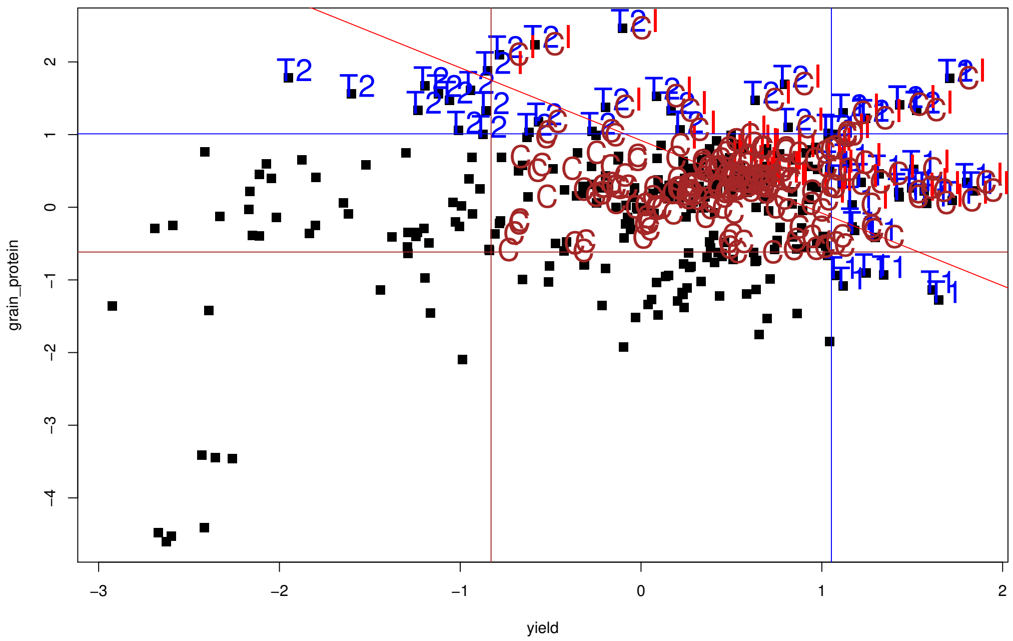

Figure 10: Barley data: Four dimensional Pareto front represented in two dimensional plots. The dimensions correspond to negative of inbreeding, and average gains based on genomically estimated breeding values for yield, height and protein content. Each point on the scatterplots correspond to a Pareto optimal solution for parental contributions. Blueness of the points measure the closeness to the ideal solution.

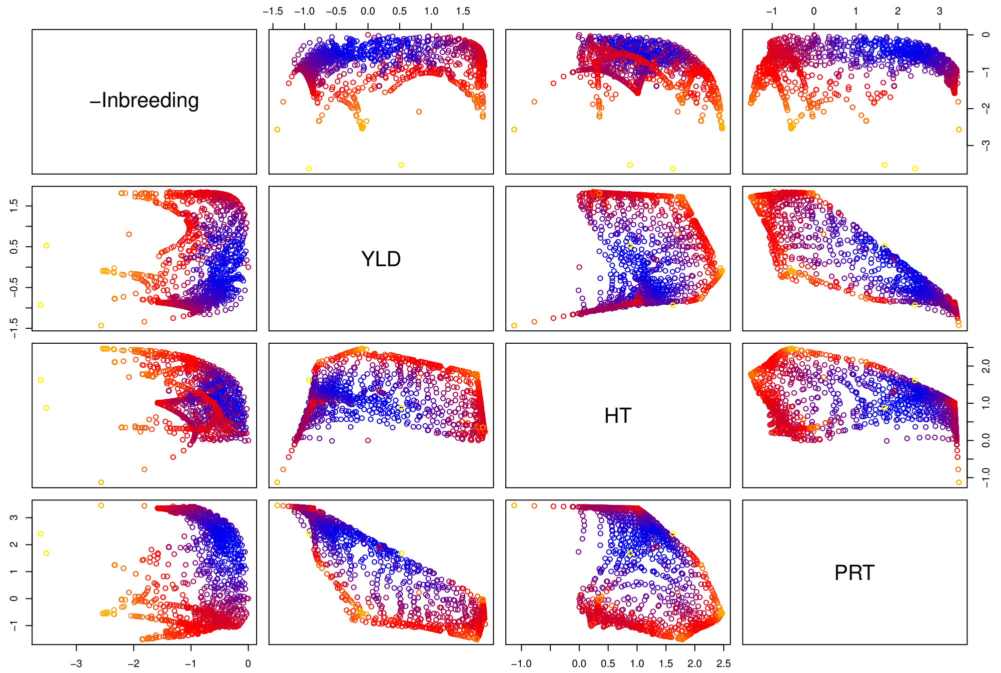

Figure 11: Barley data: SOM plot for barley data for GEBVs over three traits, yield, protein content and height. In this example, the figure with the title "Mapping" displays the mapping of the genotypes into clusters that are obtained using SOM analysis. These clusters are displayed in a two dimensional grid and the topology preserving mapping property means that closely located clusters contain genotypes with similar properties in terms of the three traits. The three dimensional dendrogram shows the closeness of these clusters in the SOM space. The change in the average values of these three traits can be observed from the "SOM Plot". In addition the surface plots display the change in the individual traits along the mapping directions of the "Mapping" plot.

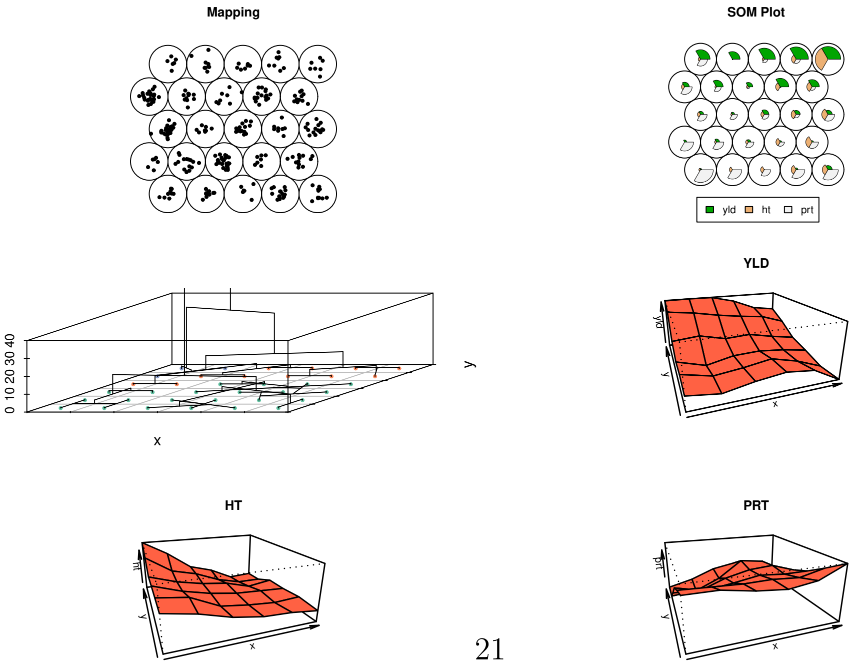

Figure 12: **Simulated data: Gain, Usefulness, Coancestry:** Genomic mating approach is also a multiobjective optimized breeding approach. The frontier surface in the figure represents the tradeoff between the measures of gain, usefulness, coancestry for pareto optimal mating plans.

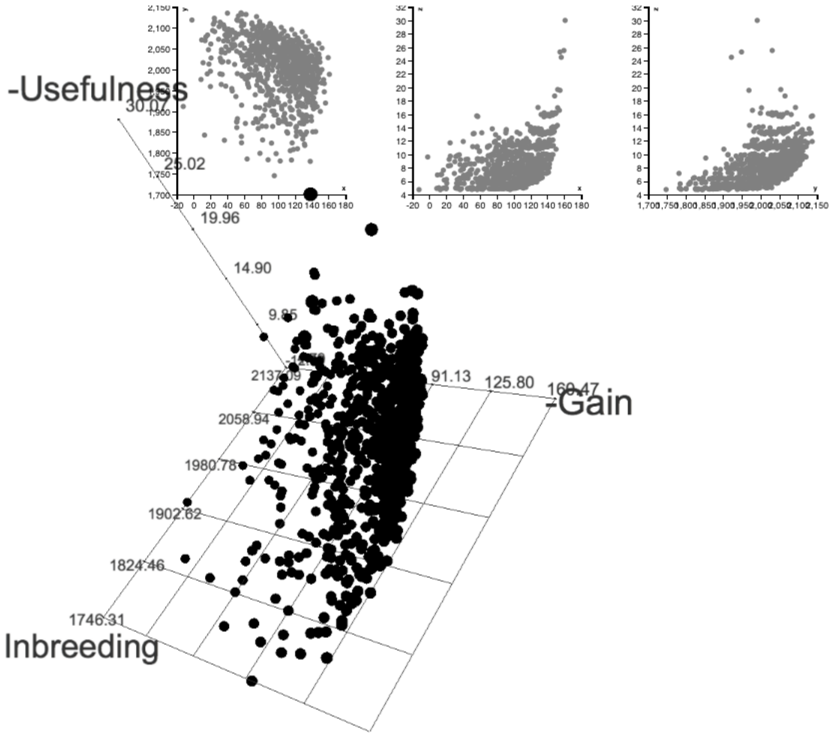

Figure 13: **Simulated data: Gain, Usefulness, Coancestry:** The top left figure "Observations overview" is a representation of all the solutions on the Pareto frontier in Supplementary Figure 12 using the mapping of these solutions into clusters that are obtained using SOM analysis. These clusters are displayed in a two dimensional grid and the topology preserving mapping property means that closely located clusters contain solutions with similar properties in terms of gain, usefulness, and coancestry. The remaining graphs are showing how each measure behaves individually on the two SOM features that correspond to the dimensions of the "Observations overview".

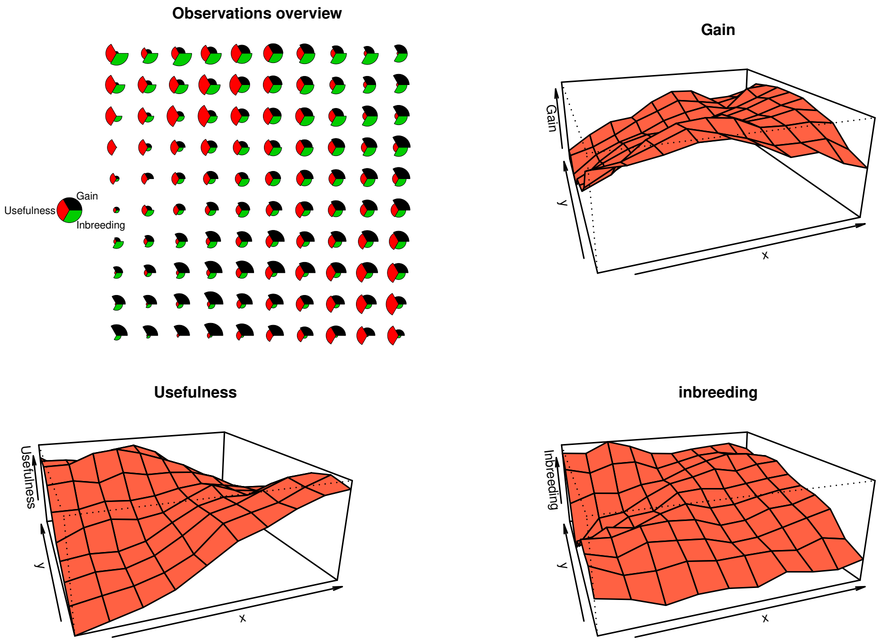

References

[1] D. Akdemir. *STPGA: Selection of Training Populations by Genetic Algorithm*, 2018. R package version 5.0.

[2] V. Chankong and Y. Y. Haimes. *Multiobjective decision making: theory and methodology.* Courier Dover Publications, 2008.

[3] J. Du and Z. Cai. A sorting based algorithm for finding a non-dominated set in multi-objective optimization. In *Natural Computation, 2007. ICNC 2007. Third International Conference on*, volume 4, pages 436–440. IEEE, 2007.

[4] C. Henderson and R. Quaas. Multiple trait evaluation using relatives' records. *Journal of Animal Science*, 43(6):1188–1197, 1976.

[5] H.-T. Kung, F. Luccio, and F. P. Preparata. On finding the maxima of a set of vectors. *Journal of the ACM (JACM)*, 22(4):469–476, 1975.

Figure 14: Self-Organizing Maps plot for barley data for parental proportions over three traits, grain yield, grain protein content and height and negative of inbreeding. This is another representation of the fourth dimensional Pareto surface in Supplementary Figure 6. The dimension x and y are found by the SOM algorithm to preserve the distances measured by the four dimensions of the frontier curve. The "Mapping" shows solutions on the frontier curve clustered with respect to x and y units (SOM dimensions), the "SOM Plot" gives the average values of the four dimensions in each cluster, the three dimensional surfaces show individual response surfaces for the four dimensions with respect to SOM dimensions.

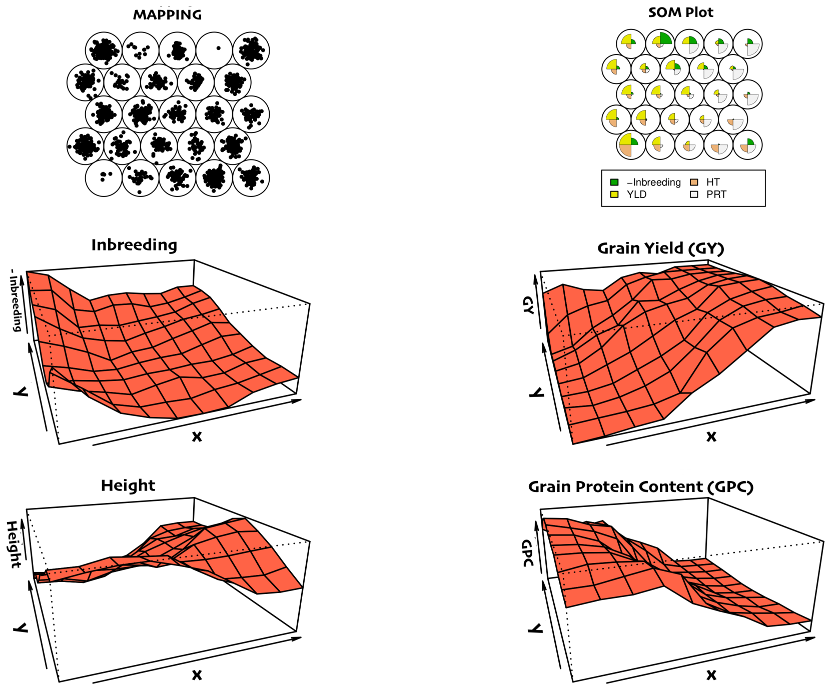

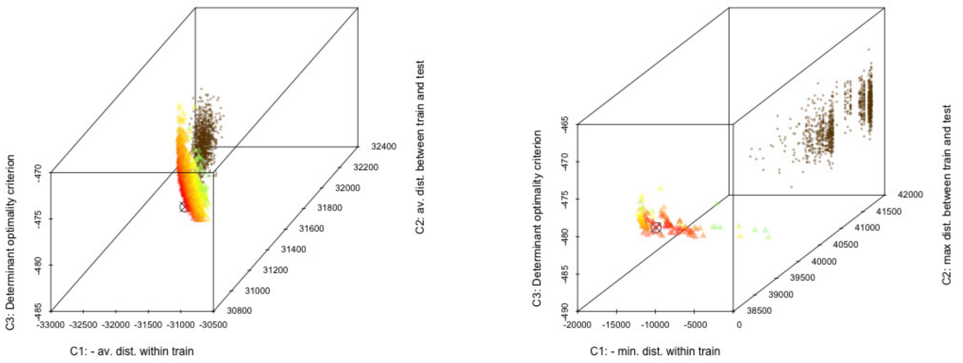

(a) (b)

Figure 15: MOO selection of training populations for Wheat data: A random subset of 50 genotypes were selected as target population. For training sets of size 100 selected from the remainder genotypes after removing the target set. The brown circles in the graphs represent criteria values for the 1000 initial solutions where the colored triangles represent criteria values for solutions on the Pareto front. The solution which has the shortest distance to the ideal solution (the solution formed by combining the best solutions for each of the criteria taken individually) is marked by a black $\otimes$ symbol. (a) Optimization Problem 1: Look for solutions that minimize DOPT criterion, negative mean genetic distance in the training population and mean genetic distance of training to target. (b) Optimization Problem 2: Look for solutions that minimize DOPT criterion, negative of minimum genetic distance between pairs of individuals in the training population and maximum genetic distance of individuals in training set to individuals in the target set.
